# Occurrence of mood disorders among educationally active older adults in Bialystok, Poland: a cross-sectional study

**DOI:** 10.1186/s12991-020-00285-4

**Published:** 2020-05-27

**Authors:** Mateusz Cybulski, Lukasz Cybulski, Elzbieta Krajewska-Kulak, Magda Orzechowska, Urszula Cwalina, Beata Kowalewska

**Affiliations:** 1 Department of Integrated Medical Care, Faculty of Health Sciences, Medical University of Bialystok, 7a M. Sklodowskiej-Curie Str, 15-096 Bialystok, Poland; 2 Faculty of Social Sciences, University of Warmia and Mazury in Olsztyn, Olsztyn, Poland; 3 Department of Statistics and Medical Informatics, Faculty of Health Sciences, Medical University of Bialystok, Bialystok, Poland

**Keywords:** Bipolar disorders, Depression, Elderly, Geriatric Depression Scale (GDS), Hypomania Check List (HCL-32), Mood disorders, Mood Disorder Questionnaire (MDQ), Older adults, Zung Self-Rating Depression Scale (Zung SDS)

## Abstract

**Background:**

Mood disorders in older people are an increasingly serious health and social problem, and their prevalence increases with age. The most common mood disorders are bipolar disorder, which is the occurrence of mania and hypomania, and depressive disorders. The aim of this study was to determine the prevalence of mood disorders in a group of educationally active elderly people living in Bialystok, Poland.

**Methods:**

The study included a total of 162 people—residents of Bialystok—aged 60 or older; 135 women (83.33%) and 27 men (16.67%). The study used five standardized psychometric scales: The Mood Disorder Questionnaire (MDQ), Hypomania Check List (HCL-32), Geriatric Depression Scale (GDS) and The Zung Self-Rating Depression Scale (Zung SDS).

**Results:**

Nearly 90.00% of the respondents obtained GDS scores indicating the presence of mild depressive symptoms; however, on the Zung SDS, which also evaluates depression symptom levels, the result obtained in almost the same number of respondents showed an absence of these symptoms. A similar percentage of respondents also obtained values on the MDQ that allow to determine a lack of bipolar disorder characteristics in the studied population. Over half of the respondents (58.02%) did not show symptoms of hypomania using the HCL-32. There was a significant correlation between the results of the GDS and Zung SDS, the HCL-32 and MDQ, as well as the HCL-32 and Zung SDS in the total studied group.

**Conclusions:**

Mood disorders, particularly depression, constitute a significant social and health problem in the group of educationally active older adults living in Bialystok. In light of the obtained research results, it is recommended to conduct and improve already realized health education programs for the elderly on the subject of mood disorder prevention and their impact on quality of life. There is a need for further research on mood disorders in the elderly to determine their prevalence on a national scale.

## Background

Aging of the population is a significant public health challenge. It is estimated that by 2050, 80% of residents in middle- and low-income countries will be people over 60 years of age. In 2020, over 1 million Poles will be 90 years old, and in 2035 over 25% will be 65 and over. In 2060, Poles will be one of the oldest societies in Europe [1]. The progressive phenomenon of an aging population around the world, and particularly on the European continent, carries a number of risks, including the mental health of seniors [2], such as mood disorders.

Mood disorders in older people are an increasingly serious health and social problem, and their prevalence increases with age [3]. The most common mood disorders are bipolar disorder, which is the occurrence of mania and hypomania, and depressive disorders [4, 5]. Bipolar disorders are described by the American Psychiatric Association’s Diagnostic and Statistical Manual of Mental Disorders (DSM-5) [6] as a group of brain disorders that cause extreme fluctuations in mood, energy and ability to function. Bipolar disorder includes three different conditions—bipolar I, bipolar II, and cyclothymic disorder. Type I bipolar disorder is a manic depressive disorder that can occur with or without psychotic episodes. Bipolar II disorder consists of depressive and manic episodes that alternate and are usually less severe and do not inhibit function. Cyclothymic disorder is a cyclic disorder that causes short episodes of hypomania and depression [6]. The average global incidence of depression among the elderly is 14.4% [7]. In Poland, on the basis of the PolSenior study, clinically significant depressive symptoms were found in 29.7% of the elderly population aged 65 and over [8]. The average incidence of bipolar disorder in patients aged 65 years and older is ~ 0.1–0.5% [9–11]. The highest prevalence in the literature was 1% among people aged 60 years or older [12]. As a result, mood disorders lead to a deterioration of the well-being and quality of life of older people, limiting independence and performance of everyday activities, and ultimately predict suicide attempts by seniors [13]. Suicides are one of the most important health problems for older people [14–16]. Although suicide attempts are more common in most countries among teenagers and young people, suicide mortality among older people is higher than in other age groups (1 in 2–4 cases) [15, 17]. The average incidence of suicide is 18–20% [18]. In relation to these data, there is a need to prevent both suicide attempts and mood disorders, which are often a predictor of their taking in the group of older adults.

Due to the increase in the senior population, there is more emphasis on active aging now. Active aging potentially enables lifelong mental well-being [19]. One of the most important elements of socially active is lifelong education. Education of the elderly is becoming the dominant topic around the world, and educational activity is a protective factor, delaying the onset of involutionary changes. It helps to mobilize intellectual and cognitive functions and to strengthen physical and mental health, and thus to life satisfaction. It creates an opportunity to acquire knowledge in various fields of science, to actively spend free time, increase the number and improve the quality of social relations, and also creates an opportunity for self-realization [20].

Despite the positive effects of education at the Universities of the Third Age described above, there is a lack of research in both Polish and world literature that identifies and describes selected mental health indicators of educationally active older adults. The vast majority of research conducted so far assesses the mental health status of elderly people with confirmed physical or mental illness, among residents of nursing homes or other similar institutions, as well as specific risk groups for older adults. To fill this gap to at least a minimum extent, the aim of this study was to determine the prevalence of mood disorders in a group of educationally active elderly people living in Bialystok. We assumed that despite the fact that the studied group consisted of socially active people, preferring active and healthy aging, the group would be characterized by an increased presence of mood disorders, particularly depression, which is one of the greater geriatric syndromes, next to dementia and delirium, together referred to as “3xD”. In addition, we assumed that the impact of mood disorders on educationally active older people will be different (more mild) than on the entire general population of seniors in Poland.

## Methods

### Participants

The study was conducted in two groups:

1. Group I—students of the University of the Healthy Senior (UHS) and the University of Psychogeriatric Prophylactics (UPP) [79 people, which included 75 women (94.94%) and 4 men (5.06%)], at the Faculty of Health Sciences, Medical University of Bialystok. The aim of the Universities is to promote a healthy lifestyle and pro-health attitudes, including in the psychological aspect; expanding knowledge in the field of: medical care improving the quality of life of the elderly, including people with mental disorders, the use of modern diagnosis and treatment methods, as well as the use of drugs (including for mental illnesses), supplements, and pharmacoeconomics in the elderly; counteracting loneliness and social exclusion and social activation of the elderly;
2. Group II—students of the University of the Third Age in Bialystok (UTA) [83 people, which included 60 women (72.29%) and 23 men (27.71%)]. The aim of the University of the Third Age (UTA) is to stimulate personal development, mental and physical acuity, social activation of the elderly, promotion of gerontological prophylaxis, and undertaking activities for the benefit of the elderly and the disabled.

The study included a total of 162 people—residents of Bialystok—aged 60 or older; 135 women (83.33%) and 27 men (16.67%). The respondents’ detailed socio-demographic data characteristics are shown in Table 1.

**Table 1:** Socio-demographic characteristics of the studied group

	UTA n = 83		UHS/UPP n = 79		Total n = 162	
	n	%	n	%	n	%
Gender						
Women	60	72.29	75	94.94	135	83.33
Men	23	27.71	4	5.06	27	16.67
Age						
60–70 years	40	48.19	56	70.89	96	59.26
71–80 years	40	48.19	22	27.85	62	38.27
81–90 years	3	3.61	1	1.27	4	2.47
Marital status						
Married	35	42.17	28	35.44	63	38.89
Widowed	33	39.76	29	36.71	62	38.27
Single	5	6.02	5	6.33	10	6.17
Divorced	8	9.64	14	17.72	22	13.58
Separated	2	2.41	3	3.80	5	3.09
Education						
Higher	45	54.22	40	50.63	85	52.47
Secondary	26	31.33	31	39.24	57	35.19
Technical	10	12.05	7	8.86	17	10.49
Vocational	2	2.41	1	1.27	3	1.85
Occurrence of mood disorders in the past						
Yes	10	12.05	11	13.92	21	12.96
No	73	87.95	68	86.08	141	87.04

*UHS* University of the Healthy Senior, *UPP* University of Psychogeriatric Prophylactics, *UTA* University of the Third Age

Additional criteria for inclusion in the study, in addition to age and place of residence, were: no diagnosed dementias and written consent for participation in the study. Each participant of the study could withdraw at any time.

Participant selection was intentional. For the study to be representative, the authors assumed collecting at least 150 fully completed questionnaires, 75 in each studied group. More copies of the research tool were distributed, however, not all of the questionnaires were returned to the authors. In the group of UHS and UPP students, 150 questionnaires were distributed (52.67% response rate), while 250 questionnaires were given out to the group of UTA students (33.20% response rate).

### Measures

#### Mood Disorder Questionnaire—MDQ

The Mood Disorder Questionnaire (MDQ) is a questionnaire for screening bipolar spectrum disorders. In addition to applications in scientific research, MDQ is above all an extremely useful tool that can be used in everyday clinical practice conditions to quickly and easily identify patients at high risk for bipolar disorder. The questionnaire allows for only a preliminary, general and working diagnosis, and people with a positive result obtained using this tool should be subjected to further, in-depth diagnosis to verify the bipolar spectrum disorder [21–23].

MDQ is a one-page self-assessment questionnaire that can be completed by the patient or a doctor, nurse or other trained medical personnel. The duration of the questionnaire is estimated to be 5–10 min. It consists of three parts:

1. A short symptom checklist, consisting of 13 questions about manic or hypomanic symptoms in the patient’s history. One can answer “yes” or “no” to each of these questions. This checklist was developed based on the criteria for mania and hypomania according to the DSM-IV classification;
2. Limited to one question about whether the symptoms listed in the checklist appeared simultaneously;
3. Aimed at assessing the degree of social functioning disorder due to the symptoms listed in the first part.

The total MDQ score is obtained by summing the “yes” answers in the symptom checklist part (the maximum score is 13 points) [21–23].

A result indicating the characteristics of bipolar disorder in an adult is at least 7 “yes” answers to questions about (hypo)manic symptoms and the occurrence of at least two of these symptoms at the same period of life. The third criterion is the answer given in the third section of the questionnaire, indicating that the described symptoms created or create a moderate or serious problem for the patient [21, 24]. The questionnaire’s sensitivity and reliability are 73.4% and 89.9%, respectively. Some data suggest that lowering the threshold of this criterion and acknowledging that it is already fulfilled when the patient assesses the symptoms presented in the questionnaire as posing a minimal problem while increasing the threshold of positive responses to the questions from the first section to at least 8 increases the tool’s sensitivity. However, this matter requires confirmation through further research [25, 26].

The MDQ alone cannot be a screening tool for the general population, but its usefulness in the study of patients suffering from mental disorders (especially affective) is indisputably very high [27].

Translation of the Mood Disorder Questionnaire into the Polish was done at the Department of Adult Psychiatry, Medical University of Poznan, with the consent of Prof. Hirschfeld [27].

#### Hypomania Check List—HCL-32

The Hypomania Check List (HCL-32) was developed by Jules Angst et al. and published in 2005. It contains a list of 32 symptoms of hypomania, plus information about the duration of symptoms, comparison with “normal” mood, and the effect of the indicated symptoms on functioning. The first version of the questionnaire was in German and was simultaneously translated into English. The English version was the basis for translation into other languages [28]. The Polish language version was created in cooperation with the list’s author at the Department of Adult Psychiatry of the Medical University of Poznan [29].

The HCL-32 is a self-assessment questionnaire. The patient independently answers questions about his/her drive, mood and activity. It can be used to self-assess the occurrence and severity of hypomania symptoms throughout the patient’s life as well as a screening tool to detect symptoms of bipolar disorder, including hypomanic symptoms, even of mild intensity in people with depression. The tool cannot be used to diagnose bipolar disorder, but it can help identify people with characteristics of bipolar disorder. The scale can be used by psychiatrists, doctors of other specialties or psychologists [28].

The HCL-32 consists of seven parts [28]:

1. The first question is used to assess whether the current mental state will affect the answers to the third question, regarding hypomania symptoms.
2. The second question is used to assess temperament, evaluating if the patient has permanent hyperthymic, cyclothymic, or depressive characteristics. This part consists of a list of questions regarding the occurrence of 32 symptoms, to which the subject has to answer yes or no. A total number of positive answers of 14 or more indicate that the respondent may have bipolar characteristics.
3. Questions 4–7 refer to the impact of these symptoms on daily life, the reactions of other people, the duration of such periods and the number of days in the last year.

A result of 14 or more points indicates the occurrence of bipolar disorder symptoms, and such persons should be accurately diagnosed by a specialist physician. Previous studies conducted using the HCL-32 showed that it is useful for assessing bipolar disorder occurrence in affective disorders. In this respect, HCL-32 has a sensitivity of 80% and specificity of 51% [28].

#### Geriatric Depression Scale—GDS

The Geriatric Depression Scale (GDS) was developed in 1983 by Yesavage et al. [30]. The full version consists of 30 items. The GDS is designed to assess depression severity over the past week in the elderly population (over 60 years of age). It is a self-assessment tool that consists of answering yes or no to short, clear questions. The questions can be read by the investigator or given in a written version to the respondent. The content deliberately omits complaints about symptoms and somatic symptoms. Individual answers are scored (0 or 1), and the obtained values range from 0 to 30 points for the full version. The results are interpreted as follows: 0–9—no depression; 10–19—mild depression; 20–30—moderate and severe depression. The scale has good psychometric properties. The sensitivity and specificity of the GDS is 84% and 95%, respectively. On average, the GDS takes 20 min to complete [30]. It has been translated into the Polish and made available for use in clinical practice by Servier Poland [31].

#### Zung Self-Rating Depression Scale—Zung SDS

The Zung Self-Rating Depression Scale (Zung SDS) is used to measure the severity of depressive symptoms in adults in the week preceding the assessment. The scale consists of 20 items. Half of the items express positive statements, scored from 1 to 4, and the other half, negative, scored from 4 to 1. The score is based on the length of time that a given symptom has been experienced over the last week. The obtained score ranges between 20 and 80. The obtained score is interpreted as follows: < 50—no depression; 50–59—mild depression; 60–69—moderate depression; > 70—severe depression. The scale has good psychometric properties as a screening tool for assessing depression. The scale’s sensitivity is 97%, and reliability 63% [32, 33]. The Zung SDS has been used as a tool to evaluate treatment results in many studies, although its limited sensitivity to change in symptom severity over time has been suggested [34]. The advantage of the Zung SDS is the ease and speed of use; on average it takes 5 min to complete. A disadvantage is the inability to assess atypical depression symptoms [35].

### Procedure and ethical considerations

The study was conducted from February to June 2018. Respondents received paper copies of the questionnaire, which they filled out at home after receiving detailed information about filling out the questionnaire from members of the study team. The research conformed with the Good Clinical Practice guidelines and the procedures were in accordance with the Helsinki Declaration of 1975, as revised in 2000 (concerning the ethical principles for medical research involving human subjects and prohibiting the provision of patient’s name, initials or hospital evidence number) and with the ethical standards of the institutional committee on human experimentation (statute from the Bioethics Committee of the Medical University in Bialystok No. R-I-002/365/2017). The members of the research team gave written and verbal information about the study to potential participants. They received the information about the project and gave written consent to participate.

### Statistical analysis

Data were prepared using Microsoft Excel 2013 and statistical analysis was done using STATISTICA 13.3 software. The following descriptive statistics were used to describe the quantitative variables: arithmetic mean, standard deviation, and median. The Shapiro–Wilk test was used to assess the normality of distribution of the quantitative variables. Normal distribution of the quantitative variables was not found. The Mann–Whitney test was used to compare two groups. Spearman’s rank correlation coefficient was used to evaluate correlations between quantitative variables. The results were considered statistically significant at p < 0.05.

## Results

The mean GDS score was 13.51 points, and thus prompted suspicion of mild depression in the studied seniors. The mean HCL-32 score was 11.20 points, which indicates a lack of hypomanic symptoms in the studied population. The mean result on the MDQ was 3.68 points, which means lack of characteristics of bipolar disorder in the elderly of Bialystok. The mean Zung SDS score was 38.63 points, which can be interpreted as the absence of depressive symptoms. The remaining values of particular variables are presented in Table 2.

**Table 2:** Descriptive statistics for individual scales

Scale	$\overline{x}$	Me	Min	Max	SD
GDS	13.51	13	6	23	3.53
HCL-32	11.20	12	0	25	6.03
MDQ	3.68	4	0	13	2.87
Zung SDS	38.63	39	20	61	9.61

GDS, Geriatric Depression Scale; HCL-32, Hypomania Check List, $\overline{x}$, mean; Max, maximum; MDQ, Mood Disorder Questionnaire; Me, median; Min, minimum; SD, standard deviation; Zung SDS, Zung Self-Rating Depression Scale

Nearly 90.00% of the respondents obtained GDS scores indicating the presence of mild depressive symptoms; however, on the Zung SDS, which also evaluates depression symptom levels, the result obtained in almost the same number of respondents showed an absence of these symptoms. A similar percentage of respondents also obtained values on the MDQ that allow to determine a lack of bipolar disorder characteristics in the studied population. Over half of the respondents (58.02%) did not show symptoms of hypomania using the HCL-32. Detailed data are presented in Table 3.

**Table 3:** The obtained point values on the individual scales, taking into account threshold values, enabled to recognize mood disorders

Scale	Point values	n	%
GDS	0–9	12	7.41
	10–19	140	86.42
	20–30	10	6.17
HCL-32	Below 14	94	58.02
	14 and more	68	41.98
MDQ	Below 7	137	84.57
	7 and more	25	15.43
Zung SDS	20–49	137	84.57
	50–59	24	14.81
	60–69	1	0.62
	70–80	0	0.00

GDS, Geriatric Depression Scale; HCL-32, Hypomania Check List; MDQ, Mood Disorder Questionnaire; Zung SDS, Zung Self-Rating Depression Scale

Over half of the respondents (n = 91, 56.17%) described their well-being on the HCL-32 as the same on the day of the study compared with their normal (typical) mood. A total of 36 people felt worse than usual (22.22%), and 35 people better than usual (21.61%). Compared with other people, a majority of the respondents (n = 96, 59.26%) stated that their activity, energy, and mood levels were always rather stable and even. Nearly ^1^/_4_ of the respondents (n = 31, 19.14%) defined their activity, energy and mood levels as better than in other people, 11 people (6.79%) as worse, while 24 respondents (14.81%) had recurring periods of highs and lows. In 57 elderly people (35.18%), close relatives reacted to or commented positively (encouragingly or supportively) about their highs. A total of 56 respondents (34.57%) described the ways others reacted or commented their good mood as indifferent, and 5 people (3.09%) felt negative reactions (in their opinion, close relatives were concerned, upset, irritable, and critical). According to 11 people (6.79%), close relatives responded to or commented about their highs both positively and negatively, while in the opinion of 33 respondents (20.37%), close relatives did not respond to their good moods at all. In the opinion of 22 respondents (13.58%), the duration of their typical highs lasted for 1 day; 45 respondents (27.78%) 2–3 days; and 15 people (9.26%) 4–7 days. Less than 10.00% of the respondents (n = 12, 7.47%) stated that their good mood lasted more than 1 week. None of the respondents indicated the answer “longer than 1 month”, while more than ^1^/_3_ of the respondents (n = 68, 41.98%) could not assess the duration of the high periods in their lives. The average number of days seniors spent in a good mood during the last 12 months was 50.62.

The majority of the respondents acknowledged that the high periods had a positive impact on family life (43.83%), social life (51.23%), and leisure (47.53%). Only in the case of professional life, respondents deemed that the high periods did not affect this aspect of their lives (49.83%). This was determined by the fact that most of the respondents did not perform any work because they were retired.

In 49.38% (n = 80) of the surveyed seniors, some of the indicated mood disorders on the MDQ occurred in the same period of life. These behaviors were not a problem for 76 respondents (46.91%); they were a small problem for 46 people (28.40%); a moderate problem for 27 people (16.67%); and a serious problem for 13 respondents (8.02%).

Analyzing the obtained mean point values in terms of age groups, the highest values on the Zung SDS were recorded in the oldest age group (71–90 years), while the highest mean point values on the GDS, HCL-32, MDQ and Zung SDS were recorded in the younger age group (60–70 years). In a comparison of age groups, it was found that the older age group (subjects over 70 years old) achieved statistically significantly higher HCL-32 scores than the younger age group (up to 70 years of age) (Table 4). Statistical analysis did not show statistically significant differences between groups of origin or the respondents’ sex. Moreover, there were no statistically significant differences between the groups in terms of marital status and education level.

**Table 4:** Comparison of descriptive values of the scales used with the respondents’ age groups

Scale	60–70 years n = 96			71–90 years n = 66			*p*
	$\overline{x}$	SD	Me	$\overline{x}$	SD	Me	
GDS	13.76	3.98	13	13.34	3.20	13	0.746
HCL-32	9.20	5.66	10	12.57	5.92	14	< 0.001*
MDQ	3.50	2.88	4	3.80	2.87	3.50	0.413
Zung SDS	39.97	9.53	40.50	37.71	9.61	37	0.152

GDS, Geriatric Depression Scale; HCL-32, Hypomania Check List; $\overline{x}$, mean; MDQ, Mood Disorder Questionnaire; Me, median; *p*, p-value; SD, standard deviation; Zung SDS, Zung Self-Rating Depression Scale

*Statistically significant value

Spearman’s rank correlation coefficients were determined between the occurrence of depressive symptoms, measured using the GDS and Zung SDS, and the occurrence of manic symptoms, measured with the HCL-32, and MDQ.

There was a significant correlation between the results of the GDS and Zung SDS, the HCL-32 and MDQ, as well as the HCL-32 and Zung SDS in the total studied group (Table 5). Taking into consideration the distribution of the studied groups according to sex, statistically significant associations between the GDS and Zung SDS, and the HCL-32 and MDQ were found among women, while between the HCL-32 and MDQ and the HCL-32 and Zung SDS in men. In the case of division into age groups, in the group aged 60–70 the coefficient between the HCL-32 and MDQ were statistically significant; while in the 71–90 age group, relationships between the GDS and Zung SDS, the HCL-32 and MDQ, and the HCL-32 and Zung SDS were significant. Detailed numerical data are presented in Table 6.

**Table 5:** Spearman’s rank correlation coefficient between the scale results in the studied group in general

	General			
	GDS	HCL-32	MDQ	Zung SDS
GDS				
r_s_	–	0.064	0.066	0.206
*p*	–	0.416	0.402	0.009*
HCL-32				
r_s_	0.064	–	0.561	− 0.265
*p*	0.416	–	< 0.001*	0.001*
MDQ				
r_s_	0.066	0.561	–	− 0.012
*p*	0.402	< 0.001*	–	0.877
Zung SDS				
r_s_	0.206	− 0.265	− 0.012	–
*p*	0.009*	0.001*	0.877	–

GDS, Geriatric Depression Scale; HCL-32, Hypomania Check List; MDQ, Mood Disorder Questionnaire; *p*, p-value; r_s_, Spearman’s rank correlation coefficient; Zung SDS, Zung Self-Rating Depression Scale

*Statistically significant value

**Table 6:** Spearman’s rank correlation coefficient between the scale results and the respondents’ sex and age

	Gender								Age							
	Women				Men				60–70 years				71–90 years			
	GDS	HCL-32	MDQ	Zung SDS	GDS	HCL-32	MDQ	Zung SDS	GDS	HCL-32	MDQ	Zung SDS	GDS	HCL-32	MDQ	Zung SDS
GDS																
r_s_	–	0.103	0.152	0.248	–	− 0.029	− 0.256	0.050	–	0.173	0.074	0.094	–	− 0.058	0.053	0.344
*p*	–	0.235	0.079	0.004*	–	0.887	0.197	0.804	–	0.091	0.472	0.362	–	0.642	0.675	0.005*
HCL-32																
r_s_	0.103	–	0.547	− 0.198	− 0.029	–	0.664	− 0.543	0.173	–	0.549	− 0.159	− 0.058	–	0.555	− 0.346
*p*	0.235	–	< 0.001*	0.021	0.887	–	< 0.001*	0.003*	0.091	–	< 0.001*	0.122	0.642	–	< 0.001*	0.004*
MDQ																
r_s_	0.152	0.547	–	0.020	− 0.256	0.664	–	− 0.211	0.074	0.549	–	− 0.010	0.053	0.555	–	− 0.014
*p*	0.079	< 0.001*	–	0.819	0.197	< 0.001*	–	0.290	0.472	< 0.001*	–	0.926	0.675	< 0.001*	–	0.909
Zung SDS																
r_s_	0.248	− 0.198	0.020	–	0.050	− 0.543	− 0.211	–	0.094	− 0.159	− 0.010	–	0.344	− 0.346	− 0.014	–
*p*	0.004*	0.021	0.819	–	0.804	0.003*	0.290	–	0.362	0.122	0.926	–	0.005*	0.004*	0.909	–

GDS, Geriatric Depression Scale; HCL-32, Hypomania Check List; MDQ, Mood Disorder Questionnaire; *p*, p-value; r_s_, Spearman’s rank correlation coefficient; Zung SDS, Zung Self-Rating Depression Scale

*Statistically significant value

## Discussion

The mean HCL-32 point value in our study was 11.20 ± 6.03, which indicates a lack of hypomanic symptoms in the studied population. Similar values were obtained by Del Carlo et al. [36], who obtained a mean HCL-32 score of 13.7 ± 5.2 in a group of 64 patients suffering from agoraphobia, and 9.1 ± 3.8 in the control group (n = 44). Values of ≥ 14 on the HCL-32 were noted in 36 subjects in the study group (56.3%) and in 4 in the control group (9.1%), whereas in our study such values were recorded in 68 respondents (41.98%). In a study by Vannucchi et al. [37], 14 or more hypomanic characteristics, verified using the HCL-32, were reported by 276 (48.3%) bariatric patients. In a group of 36.6% (n = 34) of patients in the Altınbaş et al. [38] study, there were ≥ 14 hypomania symptoms using the HCL-32. The mean HCL-32 value in the cited study was 8.1 ± 9.8.

In another study by Del Carlo et al. [39], in a group of 47 people with anxiety disorders, mean HCL-32 was 13.4 ± 5.2, while in the 45-person control group it was 9.1 ± 3.8. Values indicating the presence of hypomania were obtained in a group of 30 patients (63.8%) and 14 people from the control group (31.1%); whereas, in a study by Kim et al. [40], the mean HCL-32 point value was 17.16 ± 4.70, and thus hypomanic symptoms were observed in the studied population, taking into account the principles of interpretation of the analyzed scale. In a study by Fornaro et al. [41], the mean HCL-32 value in a group of 98 people diagnosed with advanced depression (26.70%) who scored ≥ 14 was 19.91 ± 4.85; and in a group of 182 patients (49.50%) who scored < 14, 5.86 ± 2.79. The mean HCL-32 value in the study by Vancampfort et al. [42], conducted in a group of 67 patients with bipolar disorder, was 15.80 ± 6.50. Perugi et al. [43] found 15 or more hypomanic characteristics based on the HCL-32 in almost half of the studied group (n = 253, 45.2%).

Rybakowski et al. [44], who included 1051 patients aged 18–77 years in their study, showed that the mean HCL-32 value was 10.3 ± 8.0, and 37.5% of the studied group exceeded the cut-off point for diagnosing bipolar symptoms. García-Castillo et al. [45], who conducted research in a group of 103 patients with mental disorders and 25 healthy people, showed that among patients with bipolar disorder the mean value of HCL-32 was 21.80 ± 5.05, among patients with depression 16.70 ± 4.53, those with generalized anxiety disorder 13.40 ± 6.06, and among healthy people (control group) 13.50 ± 6.12. A possible explanation for the differences in the mean point values of hypomania symptoms in our research and the cited studies by other authors may be differences in the level of medical care that the respondents were under, a low level of awareness among older people about specialist medical consultations regarding mood disorders, and the co-occurrence of other mental illnesses. In addition, cultural differences, environmental factors, and lifestyle may explain the observed differences between the individual study results using the HCL-32.

The MDQ is a short self-assessment questionnaire that was developed for screening for bipolar disorders, in particular for clinicians who often omitted the diagnosis of these disorders in people being treated for depression [46]. However, in studies in patients with mental disorders [47–49] as well as studies conducted in the general population [50, 51], the authors found low percentages of positive MDQ results. The mean MDQ score in our study was 3.68 ± 2.87, which means a lack of characteristics of bipolar disorder in the studied seniors. The percentage of patients in the study by Altınbaş et al. [38] who scored ≥ 7 points on the MDQ was 29.0% (n = 27). The mean MDQ value in the cited study was 4.1 ± 3.0.

Vöhringer et al. [52], who included 197 patients from 10 primary care centers in Santiago, Chile, in their study, found that the mean MDQ value was 4.74 ± 2.70. In a study by Zimmerman et al. [46], analysis of the MDQ results based solely on the presence of individual symptoms indicated a positive result in 131 people (27.3%), 39 of whom were diagnosed with bipolar disorder. Rybakowski et al. [44] showed that the mean MDQ value was 3.6 ± 3.2, and 20% of the studied group exceeded the cut-off point for diagnosing bipolar symptoms. In addition, the authors showed a statistically significant correlation between the MDQ and HCL-32, as well as a greater ability to identify hypomania symptoms using the HCL-32. This was also confirmed by our own research as well as studies carried out in Italy [53] and Spain [54]. A possible explanation for this may be a slightly different set of hypomania symptoms in the two questionnaires.

Symptoms of hypomania on the MDQ are a reflection of the DSM-IV criteria for mania (hypomania), while the set of symptoms in the HCL-32 is broader and more multidimensional (it pertains to many specific everyday activities that could be modified during hypomania). We noted a positive MDQ result in 15.43% of the respondents (n = 25). The result obtained in the examined group of older people was higher than in studies on adolescents. In the Wang et al. study [55], 2.0% of the studied population, i.e., 38 people from 1948 subjects, obtained a positive MDQ value. Chung et al. [50], who conducted a study on 1518 people from the general population of Hong Kong, obtained a value of 1.4%. In the Korean study by Bae et al. [56], conducted on 1020 students, a positive MDQ result was obtained in 2.3% of the respondents. Of 1002 young people in Spain, of a mean age of 21.11 years, 3.4% of the respondents had a positive MDQ result [57]. The reason for the difference between our results and those of other studies is the smaller number of participants in our study, as well as the higher risk of bipolar symptom occurrence in the elderly group.

The mean Zung SDS score in our study was 38.63 + 9.61, which can be interpreted as the absence of depressive symptoms. In the Vannucchi et al. study [37], the obtained mean severity of depressive symptoms measured with the Zung SDS was 53.2 ± 9.3. Almost identical results using the same scale were obtained by Perugi et al. [43], who obtained a score of 53.0 ± 9.0. In the FINE study conducted by Giltay et al. [58], in a group of older men from Finland (n = 716), the Netherlands (n = 887), and Italy (n = 682), the mean depression symptom severity level on the Zung SDS was 47.1 ± 10.5 among the inhabitants of Finland, 43.1 ± 9.8 the Netherlands, and 49.8 ± 11.4 Italy. Suzuki et al. [59] obtained a higher mean Zung SDS score in patients with Parkinson’s disease (43.4 ± 9.6) than in the control group (35.4 ± 8.2). Of the 188 patients with Parkinson’s disease, 122 (64.9%) showed symptoms of depression. Vélez-Álvarez et al. [60] reported symptoms of depression in 38.6%. Most of them (27.4%) had symptoms of mild depression, similarly to our study.

The prevalence of depressive symptoms in our study, using the Zung SDS, was noted in 15.43% of the respondents. Adogwa et al. [61] showed a mean Zung SDS value in a group of 69 patients at 40.43 ± 7.04. Lin et al. [62] found that in a group of 112 patients treated for advanced depression, the mean Zung SDS score before treatment was 60.3 ± 8.5 and after treatment 52.9 ± 12.1. The average assessment of depression symptoms using the Zung SDS in the study by Kamphuis et al. [63] was 42.6 ± 7.10, which means that it was lower that the cut-off value for mild depressive symptoms (50–59). The incidence of depressive symptoms in the cited study was 22.00% (14.00% mild depressive symptoms, 7.00% moderate, and 1% severe). The vast majority of the respondents (75.83%) studied by Milanović et al. [64] were in the correct range on the Zung SDS, while 19.38% (n = 149) had mild, 5.46% (n = 42) moderate, and 0.91% (n = 7) severe depressive symptoms. In a study by Gibson et al. [65], conducted in a group of 200 elderly residents of Jamaica, the mean Zung SDS score was 43.5 ± 10.5. Bove et al. [66], in a study conducted in a group of 106 older people, obtained a mean Zung SDS value of 42.8 ± 8.37.

The higher rates of depression in the above-cited studies compared with our study can be attributed to the screening tools used and the studied patient population. In other studies in the elderly population, research tools such as the Geriatric Depression Score or the Beck Depression Inventory were used. On the Zung SDS, sub-elements such as fatigue, sleep disorders or constipation could be based on symptoms of other somatic diseases. Moreover, because the Zung SDS is a self-assessment tool, there is a tendency to overestimate depressive symptoms in the elderly. Andriopoulos et al. [67] found higher Zung SDS values in women (48.22 ± 1.4) than in men (42.71 ± 1.45). Zhong et al. [68] showed an increase in depressive symptoms according to the Zung SDS in men at 45.78 ± 10.82 and women 48.94 ± 10.15. We demonstrated a reverse dependence in our study; higher mean point values on the Zung SDS scale were noted in men (41.33 ± 7.92) than in the group of studied women (38.09 ± 9.85). The reason for this could be the small size of the group of men in our study compared with the cited studies.

The mean GDS score was 13.51 ± 3.53 points, and thus prompted suspicion of mild depression in the studied seniors. Symptoms of mild depression were found among 86.42% of the respondents, and severe depression in 6.17%. Of 4352 participants of a study by Shimada et al. [69], using a shortened version of the GDS (GDS-15), 3695 people (85.00%) did not show symptoms of depression (GDS-15: 5 points), 570 respondents (13.00%) indicated the criteria for light depression (GDS-15: 6 and more points), and in 87 subjects (2.00%) depression was diagnosed. The percentage of people with coexisting symptoms of depression increased with age. In the group of people aged 65–69 it was 10.6%, and among those aged 85 and older 26.5%. A reverse correlation was demonstrated in our study; in the 60–70 age group, a slightly higher mean GDS score (13.76 ± 3.98) was observed than in the 71–90 age group (13.34 ± 3.20). This result could have been affected by the smaller sample size as well as the full version of the GDS, consisting of 30 items.

In the study by Tosangwarn et al. [70], 41.4% of residents of social welfare facilities experienced symptoms of depression, measured using the GDS-15 (n = 128). The vast majority of residents had mild symptoms, while moderate symptoms were noted in 3.9% (n = 5), and severe symptoms in 6.3% (n = 8). In the entire sample included in the study by He et al. [2], the mean GDS score was 12.45 ± 9.55 for men and 11.76 ± 9.34 for women, while 27.00% of the study participants had clinically significant depression (GDS > 10). In our study, we found slightly higher mean GDS values in women (13.56 ± 3.44) than in the group of men (13.26 ± 4.00). This could be the result of the small group of men in the studied sample.

The incidence of depression among residents of a local community in Ghana was 37.8% (23.3% mild depression, 9.2% moderate, and 5.3% severe) [71], and 49.5% among 206 elderly Nigerians [72]. Regarding depressive symptoms in the Chardosim et al. study [73], the mean score obtained on the GDS-15 was 5.17 ± 3.20, and in 43.00% (n = 13) of the participants obtained values suggesting or indicating the presence of these symptoms. In a study by Aly et al. [74], 62.7% of the respondents suffered from depression, of whom 43.8% had mild depression according to the GDS-15, and 18.9% had symptoms of moderate depression. None of the participants had severe depression. Tkacheva et al. [75] found symptoms of depression using the GDS-15 among 128 residents of Moscow over the age of 65 (36.2%). Nery et al. [76] conducted a study in a group of 140 geriatric patients in Brazil and discovered that 62.7% of the subjects did not have symptoms of depression (mean GDS score was 3.09 ± 1.26), 33.6% of the patients had mild depressive symptoms (7.51 ± 1.47), while 3.6% had severe depressive symptoms (11.20 ± 0.44).

The high rate of depression in our study compared with the studies of other authors, where lower levels of depression prevalence in older patients were found, could be caused by the fact that our study was based on questionnaires distributed without providing detailed instructions on filling the forms. The incidence rate may also be determined by different cut-off points depending on scale validation to the optimal conditions in a given country. The high percentage of depression probability among seniors revealed in this study compared with much lower rates in the cited foreign studies of the geriatric population could also be dependent on differences in the living conditions and economic situation of elderly people in Poland as well as socio-cultural factors. It is worth mentioning that on the Zung SDS, which also evaluates depression symptom levels, the result obtained in almost the same number of respondents indicated an absence of these symptoms. The reason for this could be better psychometric values of the GDS for the elderly, because it is a tool specific to this age group, while the Zung SDS can be used to examine people from every age group.

When comparing older students with younger students, the results do not differ much from those in the younger age groups. Symptoms of mild depression were found in over 85.00% of respondents, and severe depression—in over 5.00% of respondents. The prevalence of depression among Chinese medical students, estimated on the basis of a meta-analysis by Zeng et al. [77], was 29.00%. The prevalence of depression among Chinese students other fields of study, estimated on the basis of a meta-analysis by Lei et al. [78], was 23.8%. Similar results were also confirmed by other studies conducted among students [79, 80]. Our study showed that the prevalence of depression symptoms among students of the Universities of the Third Age was much higher than for young students from various universities around the world. This may be associated with various, very often negative experiences of older people during their lifetime (e.g., widowhood, retirement, divorce, etc.), compared with young students, as well as longer life expectancy, which is associated with richer life experience. Some studies have shown a higher level of depression among female students than male students [81, 82]. Similar conclusions can be demonstrated in our study, but there was no significant difference in the incidence of depression among gender. Similarly, other studies conducted among young students did not show differences in depressive symptoms between male and female students [83, 84].

The prevalence of bipolar disorder in our study was 15.4% using the MDQ and less than 42.00% using the HCL-32. Considering the results obtained using MDQ, similar results were obtained by Chanen et al. [85] and Shenoy et al. [86]. The results in the own study and the cited studies were much lower than in the publication of Zanarini et al. [87], in which almost 70.00% of respondents met the criteria for bipolar disorder. In a meta-analysis of 43 studies, conducted by Meaney et al. [88], the prevalence of bipolar disorder in college samples ranged from 0.5 to 32.1%. In our study, there was no gender difference in bipolar disorder rates, which was similar to most epidemiological studies [89, 90].

The conducted study had several limitations. First of all, although the scales used are sensitive tools for detecting mood disorders, false negatives cannot be ruled out. All scales focus on the subjective symptoms of the above disorders, and objective (clinical) criteria were not taken into account. Secondly, for the survey to be representative on a national scale, it should be extended to a larger group of older students of the Universities of the Third Age from all over Poland. Thirdly, the studied group is characterized by an overrepresentation of women in relation to men. Future studies should include a larger (comparable) number of men.

## Conclusions

In conclusion, mood disorders, particularly depression, constitute a significant social and health problem in the group of educationally active older adults living in Bialystok. In light of the obtained research results, it is recommended to conduct and improve already realized health education programs for the elderly on the subject of mood disorder prevention and their impact on quality of life. There is a need for further research on mood disorders in the educationally active elderly to determine their prevalence on a national scale.

## Data Availability

Data supporting the findings are available upon request. Please contact the corresponding author Mateusz Cybulski (mateusz.cybulski@umb.edu.pl) for data access.

## Abbreviations

DSM-IV: Diagnostic and Statistical Manual of Mental Disorders 4th ed
DSM-5: Diagnostic and Statistical Manual of Mental Disorders 5th ed
GDS: Geriatric Depression Scale
HCL-32: Hypomania Check List
MDQ: The Mood Disorder Questionnaire
UHS: University of the Healthy Senior
UPP: University of Psychogeriatric Prophylactics
UTA: University of the Third Age
Zung SDS: The Zung Self-Rating Depression Scale
